# Application of biological and green nanomaterials in wastewater treatment: techniques for the effective removal of dyes, heavy metals, and organic pollutants

**DOI:** 10.55730/1300-0152.2760

**Published:** 2025-08-27

**Authors:** Emine Sena KAZAN KAYA, Zeynep CİĞEROĞLU, Tansel KEMERLİ KALBARAN, Başak TEMUR ERGAN, Zeynep Mine ŞENOL, Meral YILDIRIM YALÇIN

**Affiliations:** 1 Department of Chemical Engineering, Faculty of Engineering, Gebze Technical University, Kocaeli, Turkiye; 2 Department of Chemical Engineering, Faculty of Engineering, Uşak University, Uşak, Turkiye; 3 Department of Nutrition and Diet, Faculty of Health Sciences, Sivas Cumhuriyet University, Sivas, Turkiye; 4 Department of Food Engineering, Faculty of Engineering, Istanbul Aydin University, İstanbul, Turkiye

**Keywords:** Wastewater treatment, biological nanomaterials, green nanomaterials, nanoadsorbents

## Abstract

**Background/aim:**

Wastewater from industrial, agricultural, and residential sources poses significant environmental and public health risks due to the presence of dyes, heavy metals, and organic pollutants. Conventional treatment methods are often inadequate for the complete removal of these pollutants. Therefore, the development of sustainable, environmentally friendly, and highly efficient treatment techniques has become increasingly important. The aim of this review is to evaluate the application of biological and green nanomaterials in wastewater treatment and to compare their effectiveness against different types of pollutants (dyes, heavy metals, and organics).

**Materials and methods:**

This review provides detailed information on the removal of various pollutants from wastewater using green and biological nanomaterials, particularly based on articles published in recent years. The review examines the structures, synthesis methods, and application areas of biopolymers, metals, metal oxides, carbon-based, and polymer-structured nanomaterials synthesized using plant extracts and microorganism-supported systems. In addition, the integration of these nanomaterials with mechanisms such as adsorption, photocatalysis, bioseparation, and membrane filtration is discussed.

**Results:**

Green and biological nanomaterials demonstrate high performance in the removal of various pollutants owing to their low toxicity, large surface area, and diverse functional groups. The synthesis of these nanomaterials using biological agents both reduces environmental impact and enhances their purification capacity. However, further research and innovation are required regarding scale-up, long-term stability, reusability, and cost-effectiveness.

**Conclusion:**

Biological and green nanomaterials represent promising alternatives for sustainable wastewater treatment. This review summarizes the current status of these materials and provides guidance for future research. Multidisciplinary approaches and expanded pilot-scale studies are essential to accelerate the transition toward industrial applications.

## 1. Introduction

Access to safe and clean water is essential for a thriving society and economy, as water is fundamental to life on Earth. Nonetheless, rapid population growth, industrialization, urbanization, and intensive agricultural practices have led to the persistent degradation of water quality, which has become a global issue (Jain et al., 2021). Rapid industrialization has resulted in the discharge of numerous contaminants into water supplies, including heavy metal ions, radionuclides, pathogenic bacteria, and viruses, thereby rendering them toxic to human health. Therefore, wastewater treatment is essential for degrading complex nondegradable pollutants present in industrial and domestic effluents, ensuring their safe discharge and preventing the contamination of surface and groundwater. The identification of wastewater pollutants is crucial for optimizing treatment processes (Katiyar et al., 2021).

**Figure 1:**
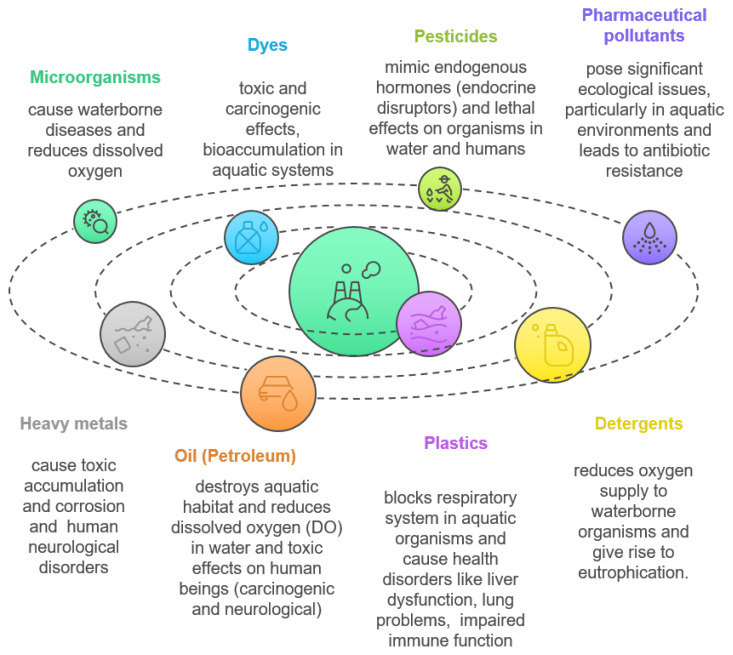
Major pollutants in wastewater and their effects on humans and the environment (Saravanan et al., 2021; Singh et al., 2020).

These pollutants can be classified based on their physical properties, chemical composition, and microbial load, and are generally categorized as organic pollutants, inorganic pollutants, radioactive pollutants, sediments and suspended solids, microplastics, microorganisms, thermal pollutants, and nutrients (Madhav et al., 2020; Jain et al., 2021; Khanam et al., 2022; Riffat and Husnain, 2022). Figure 1 illustrates the major water pollutants and their impacts on human health and the ecosystem (Singh et al., 2020; Saravanan et al., 2021).

Wastewater treatment (WWT) is the process of removing pollutants and contaminants through physical, chemical, or biological methods before its release into the environment, aiming to recover water and micronutrients and to mitigate risks to public health and the environment (Jain et al., 2021). Wastewater treatment often involves the sequential application of multiple procedures; therefore, the selection of treatment techniques is influenced by these conditions. The first consideration is the treatment level required to ensure that wastewater quality meets permissible standards. Flexibility in the control method is also important, allowing the process to adapt to changing conditions. Additionally, the cost of the treatment process must be assessed to ensure economic viability. Lastly, environmental compatibility is a crucial factor, requiring that the chosen method minimizes environmental impact and aligns with sustainability objectives.

Wastewater treatment techniques can be categorized into chemical, physical, and biological processes. Physical methods include comminution, screening, grit removal, dissolved air flotation (DAF), sedimentation, ion exchange, adsorption, and membrane filtration. The primary objective of physical treatment techniques is the removal of suspended solids. Biological treatment is classified according to oxygen availability into anaerobic and aerobic processes, in which microorganisms degrade organic pollutants. The objectives of biological treatment include the removal of nutrients such as nitrogen and phosphorus from wastewater, adjusting biodegradable organic matter to levels that comply with regulatory standards, and preventing problems such as eutrophication in receiving water bodies. Chemical treatment in wastewater management is a critical process that employs chemical reactions to convert, neutralize, or eliminate contaminants, thereby ensuring compliance with environmental discharge standards. Fundamental techniques include coagulation-flocculation for removing suspended solids, disinfection for eliminating pathogenic microorganisms, and chemical precipitation for reducing phosphorus levels. Furthermore, advanced chemical processes are applied to effectively target and remove a broad range of pollutants. Collectively, these methods enhance the efficacy and reliability of wastewater treatment, contributing to the production of effluent that complies with stringent environmental regulations (Saravanan et al., 2021; Riffat and Husnain, 2022; Kato and Kansha, 2024). The selection and combination of wastewater treatment methods are primarily determined by the specific characteristics of the wastewater and the advantages and limitations of each technique, as presented in Table S.

However, these conventional methods have several limitations, including the incomplete removal of recalcitrant pollutants, high operational costs, and the need for expensive chemical reagents. To address these challenges, the integration of nanomaterial-based techniques has gained attention as a forward-looking approach, offering enhanced contaminant removal efficiency and contributing to the overall improvement of wastewater treatment processes. Nanomaterials or nanoparticles (NPs) significantly enhance adsorption capacity in wastewater treatment owing to their high surface area-to-mass ratio, which provides numerous active sites for contaminant interaction and removal.

Green nanomaterials are synthesized through green methods using natural, eco-friendly precursors that are safe for both ecosystems and human health. Green nanotechnology focuses on producing nanomaterials from natural and biodegradable raw materials. This approach integrates nanotechnology with green chemistry principles to develop sustainable solutions for WWT (Singh et al., 2020; Neeti et al., 2023). Biological nanoparticles represent another alternative for wastewater treatment owing to their eco-friendliness, nontoxic nature, and ease of production through simple green synthesis routes. Therefore, biologically driven nanotechnology limits the risk of forming toxic intermediates and final products. Moreover, this approach offers significant advantages for both human health and environmental sustainability (Sati et al., 2022; Karki et al., 2023). Numerous in-depth studies have been published, highlighting the diverse applications of various nanomaterials in WWT. However, a comprehensive and specialized review addressing the specific role of biological and green nanomaterials in wastewater treatment is still lacking in the literature. Therefore, the aim of this review is to provide a critical evaluation of the application of biological and green nanomaterials in wastewater treatment, with an emphasis on their effectiveness in removing pollutants and contaminants.

## 2. Nanomaterials in wastewater treatment technology

In nanotechnology, materials are studied, analyzed, and applied at the nanoscale, where atoms and molecules exhibit behaviors distinct from their bulk counterparts. The tunable pore volume, high specific surface area, antimicrobial activity, hydrophilic and hydrophobic properties, and electrostatic interactions of nanomaterials have enabled their application in adsorption, photocatalysis, and the removal of various pollutants and contaminants from wastewater. According to the literature, nanomaterials are categorized based on their production methods, chemical composition, research and industrial applications, dimensions and features, and origin. Therefore, nanomaterials can be classified in various ways. This review focuses on nanomaterials used in wastewater treatment applications.

**Figure 2:**
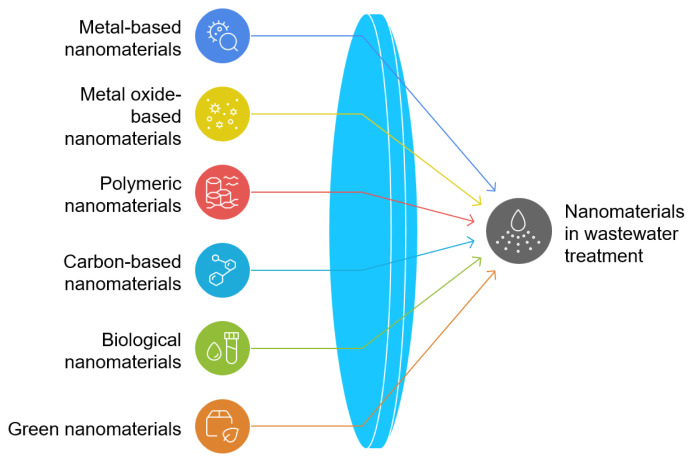
Classification of nanomaterials used in wastewater treatment technology.

Traditional methods often face challenges, including incomplete pollutant removal, the generation of secondary waste, and high operational costs. The emergence of nanotechnology, particularly metal-based nanomaterials (iron, silver, titanium, and zinc), offers innovative solutions to these challenges. Due to their exceptional physical and chemical properties, nanomaterials are highly attractive for various wastewater treatment applications. For example, metallic nanoadsorbents such as zinc oxide (ZnO), titanium dioxide (TiO_2_), aluminum oxide (Al_2_O_3_), and iron oxides have been extensively investigated for the removal of metals from wastewater due to their affordability and effectiveness. In addition to their exceptional ability to eradicate harmful microorganisms, ZnO nanoparticles can also be applied in UV disinfection to improve water quality (Neeti et al., 2023). Pd nanoparticles and silver-based nanocatalysts have been shown to enhance the biodegradation of halogenated organic compounds and the degradation of organic pigments. Hematite (α-Fe_2_O_3_), magnetite (Fe_3_O_4_), and maghemite (γ-Fe_2_O_3_) are the three main types of magnetic nanoparticles, all of which are effective nanoadsorbents. Superparamagnetic iron oxides are useful for the removal and separation of metal impurities. Metallic and magnetic nanoadsorbents represent just two examples. Various types of nanomaterials, as shown in Figure 2, have been studied for purposes such as disinfection, pollutant degradation, inorganic remediation, and antibacterial activity, which will be discussed in detail in this review.

### 2.1. Metal-based nanomaterials

Metal-based nanomaterials exhibit enhanced reactivity, large surface areas, and the ability to target specific contaminants, making them ideal candidates for advanced WWT. For example, silver nanoparticles possess antimicrobial properties and can efficiently inactivate bacteria, viruses, and other pathogens in wastewater. Iron nanoparticles are widely employed for pollutant removal through reduction and adsorption processes. They can effectively reduce toxic metals such as chromium and lead into less harmful forms (Thilakan et al., 2022). Simple inorganic titanium (Ti) nanoparticles are effective in removing turbidity, organic matter, and heavy metals. However, Ti nanoparticles exhibit several shortcomings, including low stability in wastewater treatment. Some studies have reported that Ti nanoparticles perform better in removing organic matter only under acidic conditions (Gan et al., 2021). Owing to their wide band gap and physicochemical stability, zinc sulfide (ZnS) and titanium dioxide (TiO_2_) semiconductors are frequently used as photocatalysts in water purification. The literature reports that TiO_2_/ZnS-based ternary nanomaterials exhibit outstanding performance in accelerating the removal of organic pollutants from wastewater (Kanakaraju and Chandrasekaran, 2023). Related studies have also documented the drawbacks of individual TiO_2_ and ZnS photocatalysts, including their limited adsorption capacity and inefficient utilization of visible light, which slow down the photodegradation of organic pollutants. These limitations can be addressed by incorporating metals and nonmetals to create TiO_2_/ZnS-based nanomaterials with tailored physicochemical characteristics to enhance photocatalytic activity.

### 2.2. Metal oxide-based nanomaterials

Metal oxide-based nanomaterials exhibit unique characteristics resulting from the combination of multiple physical and chemical properties. In particular, the integration of mechanical, optical, electrical, thermal, and chemical properties provides these materials with multifunctionality and broad potential for wastewater treatment. A key feature of metal oxides and their composites is the ease with which their size, shape, and surface chemistry can be tailored. These properties make them highly effective and selective adsorbents, particularly for the removal of heavy metal and metalloid ions such as arsenic, lead, mercury, and cadmium from wastewater. Compared to other nanostructures, nanoporous metal-based nanomaterials are more applicable owing to their abundant active sites, nanopores/mesopores, and physicochemical characteristics. However, Baranwal et al. (2018) reported that among metal and metal oxide nanoparticles, silver (Ag) nanoparticles have been used on a much larger scale than other types due to their microbicidal effect. Moreover, ZnO and TiO_2_ possess photocatalytic properties that enable the degradation of organic pollutants under UV light (Kanakaraju and Chandrasekaran, 2023). Their stability and nontoxicity render these materials suitable for diverse wastewater treatment applications. Iron oxides (Fe_2_O_3_, Fe_3_O_4_) are effective adsorbents for heavy metals and can also facilitate contaminant reduction through redox reactions. Recent studies have reported an increasing number of publications on the use of iron oxides, Fe_3_O_4_ nanoparticles, and magnetite nanoparticles in WWT, along with research on biological and green synthesis methods (Jabbar et al., 2022). Cerium oxide (CeO_2_) can aid in the removal of reactive oxygen species (ROS) and has potential applications in industrial wastewater treatment. Applications of CeO_2_ nanoparticles in WWT include advanced oxidation processes, adsorption, and filtration (Kurian, 2020; Farooq et al., 2024). In addition, CeO_2_ and its composites have exhibited promising outcomes in photocatalysis, sonocatalysis, and electrocatalysis for the degradation of organic pollutants in wastewater. For instance, CeO_2_ is a strong oxidizer that generates radicals for a variety of pollutants. Under visible light, Ce^3+^ forms an electron–hole pair, which can interact with OH^−^ ions or H_2_O to produce ^•^OH radicals that play a critical role in Fenton reactions (Ali et al., 2024). Moreover, rare earth oxide-mixed cerium-based metal oxide nanocomposites have shown great promise in these areas in recent years. For example, NiO-CYSO nanomaterials were synthesized using a wet chemical process for multiple applications owing to their photocatalytic and antibacterial properties (Kannan et al., 2020). Another study focused on synthesizing hexagonal cesium (Cs)-doped molybdenum trioxide (MoO_3_) nanostructures (NSs) for potential application as catalytic and antibacterial agents against methylene blue dye and multidrug-resistant (MDR) E. coli (Ikram et al., 2024). In addition to its exceptional capacity to adsorb organic pollutants and metallic elements at high density, copper oxide (CuO) exhibits antimicrobial and photocatalytic properties. The superior solar-driven photocatalytic performance of CuO results from its efficient utilization of UV radiation; however, its narrow band gap may lead to electron–hole recombination.

### 2.3. Polymeric nanomaterials

Polymeric nanomaterials (nanofibers, nanoparticles, hydrogels, and composite materials), characterized by their nanoscale dimensions and versatile chemical properties, have gained attention as advanced materials for enhancing wastewater treatment processes such as filtration, adsorption, catalysis, and biodegradation. Among polymeric nanomaterials, membrane technology offers significant potential for wastewater treatment due to its low energy consumption, compact size, and low initial cost. Polymer nanocomposites can be categorized into distinct groups based on their application in wastewater treatment, including photoresponsive polymer-based nanomaterials, multifunctional polymer-based nanocomposites, and adsorptive polymer-based nanocomposites (Hazarika et al., 2023). The most common application of polymer-based nanocomposites in wastewater treatment is membranes, which serve as selective barriers by retaining pollutants on the surface while allowing desired substances to pass through their pores. Polymer nanocomposite (PNC) membranes, which can be described as nanoparticle-embedded membranes, are highly permeable, selective, and cost-effective for efficient wastewater treatment. PNCs can be synthesized using various methods, including the sol–gel method, interfacial polymerization, phase inversion, physical mixing, chemical grafting, coating deposition, track etching, stretching, self-assembly, electrospinning, and layer-by-layer assembly (Nath et al., 2024). Zheng and colleagues investigated a polysaccharide-based polymer nanocomposite for wastewater treatment. Polysaccharides are typically derived from plants. Due to their hydrophilic functional groups, polysaccharide-based nanocomposites can adsorb water even though they are soluble at their natural pH (Zheng et al., 2015). Chitosan, another important linear polysaccharide, is produced by the deacetylation of chitin derived from algae, fish, insects, fungi, shrimp, and other sources. Chitosan-based nanocomposites can efficiently remove heavy metal ions without the aggregation and gelation problems associated with chitosan (Hazarika et al., 2023). Natural polymer-based nanocomposites have shown positive results in the removal of organic contaminants from effluents as well as from surface and groundwater. In addition, other polymer-based nanocomposites have been increasingly applied in wastewater treatment in recent years. Examples include ZnO and poly(2-hydroxyethylmethacrylate)–graphene oxide hybrid nanocomposites, which were used to remove sodium lauryl sulfate, paracetamol, and dyes from water (Ussia et al., 2018). MnO_2_/Fe_3_O_4-_based polyaniline nanocomposites were synthesized by depositing MnO_2_ and Fe_3_O_4_ on the surface of polyaniline microspheres for dye removal (Dutta et al., 2021). A graphene-based polypyrrole nanocomposite was investigated for the removal of perchlorate contamination (Zhang et al., 2011).

### 2.4. Carbon-based nanomaterials

A wide range of carbon-based nanomaterials, such as fullerenes (0D), carbon nanotubes (CNTs) (1D), and graphene and graphene oxide (2D), have been studied to enhance the adsorption capacity of conventional adsorbents. In addition to their chemical stability and ability to adsorb a wide range of pollutants, these materials are of great interest due to their high surface area. According to studies, the shape, size, and diameter of CNTs influence their efficacy in degrading and/or adsorbing pollutants. They also exhibit strong dependence on pH and contact time (Mittal et al., 2015).

Graphene’s extremely high conductivity and mechanical strength make it a unique material (Kaya and Oktem, 2025). Graphene has been applied in pretreatment studies utilizing its sedimentation properties, in secondary treatment with coagulants, and in tertiary treatment involving purification techniques such as membrane filtration, adsorption, depth filtration, gas stripping, and anaerobic sludge treatment (Suárez-Iglesias et al., 2017). Graphene oxide, owing to its functional groups, enhances interaction with pollutants, making it suitable for the removal of dyes, heavy metals, and pharmaceuticals from wastewater. The carbon allotrope fullerene consists of closed, hollow carbon cages. For example, fullerene C_60_ acts as a photosensitizer that generates reactive oxygen species (ROS) by transferring energy or electrons to ground-state oxygen in wastewater. These ROS function as oxidants, breaking down various organic compounds and disinfecting microorganisms (Taghipour et al., 2019). However, it is important to emphasize that before applying nanotechnology in wastewater treatment, several factors must be considered, including effluent quality criteria, nanomaterial effectiveness, recyclability, ecological impact, and cost. The primary source of carbon for carbon-based nanomaterials is hydrocarbons derived from fossil fuels. Moreover, despite their remarkable applications, the production of some carbon nanomaterials can be hazardous, and certain types may even exhibit toxicity. Therefore, researchers have focused on the synthesis of carbon materials through green approaches (Temur Ergan et al., 2023). This review summarizes different techniques used in wastewater treatment, with a focus on the development and application of sustainable tools in WWT, such as environmentally friendly nanoparticles, including biological and green nanomaterials.

### 2.5. Biological nanomaterials

Despite advancements in nanotechnology, there is still a lack of research on the potential effects of biological nanomaterials on human health and environmental well-being. If treatment techniques are inadequate, biological nanomaterials may pose environmental risks (Saud et al., 2024). It is now recommended to employ green nanoscience to reduce the potential hazards associated with the production and use of biological nanomaterials and to promote the development of more environmentally friendly and advanced nanomaterials. Biobased nanomaterials, a viable substitute for synthetic nanomaterials, offer advantages such as sustainability, biocompatibility, affordability, and biodegradability. Biobased nanomaterials are typically derived from biological processes and living organisms. These resources contain biologically active compounds such as polyphenols, enzymes, flavonoids, proteins, and terpenoids, which act as reducing agents, catalysts, and stabilizers. Therefore, they can be classified as bacterial nanomaterials (biosurfactants, bacteria-derived nanoparticles), enzymatic nanomaterials (enzyme-immobilized nanoparticles, laccase nanoparticles), and biochar nanomaterials derived from biomass. Biomass-derived biological nanomaterials hold great potential for use in current WWT. Biochar derived from biomass is a carbon-rich material capable of adsorbing various contaminants and improving the overall quality of treated wastewater. The effectiveness of these materials can be enhanced by modifying their surface properties through various chemical and physical methods (Temur Ergan et al., 2023). However, when evaluating the benefits of biomass-derived materials, the energy costs associated with their reprocessing and recycling must also be considered. Biosurfactants are microbial surface-active agents produced by bacteria. These compounds can emulsify and degrade hydrophobic pollutants in wastewater systems. Certain bacteria-derived biological nanoparticles can synthesize metal nanoparticles with antimicrobial properties, thereby aiding in the treatment of contaminated water (Saud et al., 2024).

Enzyme-immobilized nanoparticles can be anchored onto biological nanomaterials to enhance the elimination of organic pollutants in wastewater through physical methods (encapsulation, entrapment, and adsorption) as well as chemical approaches. The combined action of enzymatic reactions and pollutant adsorption on solid supports such as graphene makes immobilized enzymes more efficient than free enzymes in removing phenolic compounds, dyes, and related pollutants. In particular, the laccase enzyme is employed for the oxidation of phenolic compounds, thereby improving the treatment of industrial wastewater. The use of carbon nanotubes as solid supports has resulted in immobilized enzymes with higher specific activity and availability than free enzymes, demonstrating their potential for industrial applications. Carbon nanotubes have been demonstrated to provide an effective support for immobilizing various wastewater treatment-related enzymes, including lipase and laccase (Xu et al., 2015).

### 2.6. Green nanomaterials

Numerous studies have demonstrated the detailed characterization and morphology of nanomaterials using a variety of biological, chemical, and physical methods. However, current chemical and physical techniques for producing nanomaterials are costly and generate highly hazardous byproducts. Another significant drawback of traditional nanoparticle synthesis methods is the requirement for extremely high temperatures and hazardous chemicals. To address these issues, the principles of green chemistry must be applied to this rapidly developing field. Consequently, research has increasingly focused on the production of green nanomaterials. Green nanomaterials are generated from natural sources such as plants, microorganisms, and various biowaste products, including fruit peels, eggshells, vegetable waste, and agricultural residues, and are designed for effective wastewater treatment. These materials are inexpensive, simple, safe, nontoxic, and environmentally friendly, representing a key element of green technologies aimed at environmental remediation and the conversion of excess bioactive materials into more economically viable and ecologically acceptable forms (Neeti et al., 2023). Green nanomaterials can be categorized into several groups, including plant-based nanomaterials, cellulose nanocrystals, algal nanomaterials (algal biomass, exopolysaccharides), and fungal nanomaterials (chitosan nanoparticles, mycelium-based nanomaterials). The use of bacteria, fungi, and algae as sources for nanoparticle synthesis is becoming increasingly popular, despite certain drawbacks, due to their ability to generate distinct nanoparticles with specific properties and applications. Biological systems such as bacteria, plants, algae, yeast, and fungi have been employed for the synthesis of metal and metal oxide nanomaterials. There are several principles that a biological nanomaterial must fulfill to be considered a green nanomaterial, as follows:

1. Little or no toxic waste should be generated during the synthesis of the nanomaterial,
2. Green nanomaterials should possess the same or superior characteristics compared to those produced by chemical methods,
3. Water should be used as the solvent during synthesis,
4. Synthesis conditions should preferably be carried out at room temperature and atmospheric pressure,
5. Plant extracts and culture media should be reusable.

In green synthesis, the plant extracts or culture media used should be easily disposable without causing environmental harm. The simplest methods for producing polymer nanocomposites include solution casting and other uniformly dispersed filler techniques. However, studies are ongoing to make these methods more cost-effective and environmentally friendly so that they qualify as green techniques. Electrospinning, freeze-drying, and irradiation methods (UV and microwave) are among the green techniques currently under study. Green nanomaterials can be produced using plants, bacteria, fungi, and other biological materials under moderate pH, temperature, and pressure conditions, and at significantly lower cost (Sanni et al., 2024). Consequently, some nanomaterials are of biological origin but do not qualify as green technology products; the distinction between the two is summarized in Table 1.

## 3. Applications of biological nanomaterials in wastewater treatment technology

Conventional treatment methods are generally insufficient for the removal of persistent pollutants. Therefore, biological nanomaterials have attracted attention as a potential solution. Biological nanomaterials are nanoscale materials derived from natural sources or synthesized through biotechnological methods. These materials may consist of natural biopolymers or nanoparticles synthesized by microorganisms. Biological nanomaterials are primarily characterized by a high surface area-to-volume ratio, biodegradability, and low toxicity (Mondéjar-López et al., 2024). Since these nanomaterials generally contain functional groups such as −COOH, −NH_2_, and −OH, they exhibit high affinity for pollutants. These materials function by effectively removing pollutants from wastewater, accelerating degradation processes, and preventing microbial contamination. Table 2 summarizes the types and properties of biological nanomaterials commonly used in wastewater treatment. Biological nanomaterials offer environmentally friendly, effective, and economically viable alternatives for WWT.

Biological nanomaterials are environmentally friendly, biocompatible, and biodegradable, and their high surface area enables the selective adsorption of pollutants. Although green synthesis methods require less energy and fewer reagents, these materials may be unstable under certain conditions, involve high production costs, and pose challenges for large-scale industrial application. Remarkably, bacteria and fungi have been shown to synthesize gold (Au), silver (Ag), copper (Cu), zinc (Zn), and platinum (Pt) nanoparticles. As shown in Table 3, a variety of species are currently utilized to produce green nanoparticles (NPs).

### 3.1. Nanobiopolymers

Nanocellulose, nanochitin/nanochitosan, and nanoalginate, which are nanostructured forms of biopolymers, are widely used in WWT. Nanocellulose is a biopolymer nanomaterial obtained by reducing cellulose to the nanometer scale (5–100 nm). It can be produced from plant-derived sources or bacterial processes. It is a renewable, biodegradable, nontoxic, and environmentally friendly biopolymer with a very high surface area, excellent mechanical strength, and strong surface functionalization potential. In WWT, nanocellulose is used for the removal of heavy metal ions and dyes, as well as for enhancing the strength and permeability of filtration membranes as a supporting material. Numerous studies in the literature have reported on the application of nanocellulose in WWT. Kawady and colleagues demonstrated that a modified nanocellulose-based biosorbent was effective for uranium (VI) adsorption, as confirmed by kinetic and thermodynamic studies. In this study, the maximum removal capacity was reported as 150 mg g^−1^ at room temperature (Kawady et al., 2022). Similarly, Jakka et al. investigated the effectiveness of nanocellulose derived from sweet lemon peel biomass for the removal of safranin O dye (Jakka et al., 2025). In this study, 99% removal efficiency was achieved within 150 min, and the maximum removal capacity was reported as 97.7 mg g^−1^. Ahmad et al. (2023) highlighted the potential of cellulose-based aerogels as sustainable and effective materials for removing heavy metals and colored pollutants from wastewater.

### 3.2. Nanochitin / nanochitosan

These materials are nanometer-sized forms of natural polysaccharides obtained from the exoskeletons of shellfish. Chitosan is produced through the deacetylation of chitin. These biopolymers are notable for their high biocompatibility, active functional groups (particularly −NH_2_ and −OH), environmentally friendly nature, and strong adsorption capacity (Khajavian et al., 2022). These biopolymers are biodegradable and environmentally safe; their surfaces can be easily functionalized, they exhibit high selectivity and adsorption capacity for metal ions, and they can be readily integrated into composite structures. The literature contains numerous studies on the use of nanochitin/nanochitosan in WWT. Atheena et al. (2024) reported that nanochitin/nanochitosan possesses unique chemical and physical properties due to its small particle size, which results in a high surface area, thereby increasing the reactivity of functional amino and hydroxyl groups and making it more effective than other materials in removing pharmaceuticals and personal care products from water. In another study, Kurniawidi et al. (2022) synthesized and characterized nanochitosan from vannamei shrimp shells. FTIR analysis revealed that the material obtained from shrimp shells initially transformed into a chitin structure, which was confirmed by the appearance of the C–O–C functional group in the spectrum. Subsequently, chitosan formation was confirmed by the prominence of O–H and N–H functional groups. SEM images showed that nanochitosan particles with an average size of 173.71 nm were successfully synthesized. The synthesized nanochitosan exhibited an 81.35% removal capacity for Fe ions from solution. Sheikhi and Rezaei (2021) investigated the removal of Cr(VI) from aqueous solutions using nanochitin. They reported that the optimum conditions were pH 6.0 and 25 °C, with a maximum removal capacity of 238.095 mg g^−1^ within 60 min.

### 3.3. Nanoalginates

Nanoalginate is the nanoscale form of alginic acid derived from brown seaweed. This environmentally friendly natural polysaccharide is water-soluble, biodegradable, and nontoxic. It can cross-link, form gels, and effectively retain pollutants, primarily due to its carboxyl (−COOH) and hydroxyl (−OH) groups (da Silva Alves et al., 2021). Numerous studies in the literature have reported on the application of nanoalginate in WWT. Almutairi et al. (2021) investigated the removal of Cr(VI) ions from wastewater using a chitosan/alginate nanocomposite combined with iron nanoparticles. They reported a maximum removal efficiency of 98.93% under optimal conditions: pH 5.0, an adsorbent dose of 4 g L^−1^, a contact time of 210 min, and an initial Cr(VI) concentration of 75 ppm.

### 3.4. Biologically synthesized nanomaterials

In this context, silver nanoparticles (Ag NPs) have received considerable attention. Silver nanoparticles can be synthesized through environmentally friendly methods using plant extracts, microorganisms, or biopolymers. This approach is environmentally safer as it eliminates the use of harmful chemicals and enables an ecological and nontoxic production process. Mohammed (2019) investigated the use of Ag NPs as disinfectants in wastewater treatment. The disinfection efficiency was evaluated against Escherichia coli by directly adding silver nanoparticles to wastewater. The results revealed that Ag NPs exert a strong bactericidal effect and can be used as potential disinfectants in wastewater treatment. In another study, Liu et al. (2021) reported that Ag NPs were adsorbed onto a mesoporous silica matrix and applied for the removal of ciprofloxacin from wastewater. The findings showed that the Ag NP-silica composite exhibited high adsorption capacity, achieving up to 98% removal of ciprofloxacin from wastewater.

Fe_3_O_4_ nanoparticles, also known as magnetite-based iron oxide nanoparticles, are environmentally friendly and reusable inorganic nanomaterials with magnetic properties, characterized by high surface area and high reactivity due to their nanoscale structure (Jabbar et al., 2022). In addition, their easy separation using a magnetic field makes them a versatile tool in wastewater treatment. Fe_3_O_4_ nanoparticles enable the removal of heavy metal ions and cationic/anionic dyes in WWT, facilitate photocatalytic degradation, exhibit antibacterial activity (particularly when coated with Ag or ZnO), and support microorganism or enzyme immobilization in biological treatment. In another study, Hassaan et al. (2021) synthesized both green and chemically produced ZnO NPs using *Gelidium pulchellum* extract as a biological reducing agent. Then they investigated their effectiveness in removing Congo red dye from wastewater. They reported that green ZnO NPs were more effective in dye removal than chemically synthesized ZnO NPs, achieving complete decolorization of Congo red (100%) within 20 min.

TiO_2_-biocomposites are hybrid materials formed by combining TiO_2_ nanoparticles with natural biomaterials such as chitosan, cellulose, alginate, and lignin. Superior performance in WWT is achieved by combining the strong photocatalytic activity and chemical stability of TiO_2_ with the renewable, biodegradable, and functional group-rich structures of biomaterials. TiO_2_ induces the degradation of organic pollutants such as dyes, pharmaceuticals, and phenols through photocatalysis. TiO_2_ exhibits antibacterial activity by generating reactive oxygen species (ROS) during photocatalytic degradation (Schnitzler and Zarbin, 2004). It also plays an active role in heavy metal adsorption and serves as a composite component in reusable membranes resistant to clogging in treatment systems. Bionanocomposites are hybrid materials formed by combining one or more biologically derived polymers (such as chitosan, alginate, cellulose, lignin, and proteins) with one or more nanostructures (e.g., metal/metal oxide nanoparticles, nanotubes, graphene derivatives). These structures are biodegradable, environmentally friendly, rich in functional groups, and possess a high specific surface area (Mittal, 2011). Bionanocomposites are applied in WWT, particularly in the removal of organic pollutants, heavy metal adsorption, microbial treatment, and photocatalytic degradation. Several studies in the literature have reported on the use of green-synthesized bionanocomposites in WWT (Rami et al., 2022).

Biological nanomaterials can be synthesized by methods such as green synthesis (biosynthesis), microbial synthesis, and in situ polymerization. In the biosynthesis method, nanoparticles are produced through environmentally friendly processes using microorganisms such as bacteria, fungi, algae, or plant extracts. This method is both environmentally friendly and cost-effective. In microbial synthesis, nanoparticles are generated using bacteria, fungi, and algae (Tauseef et al., 2023). For example, Ag NPs synthesized by the bacterium *Shewanella oneidensis* exhibit antibacterial properties and contribute to pathogen removal in wastewater treatment (Rajput et al., 2021). In the in situ synthesis method, nanoparticles are directly formed within the biopolymer matrix (Adnan et al., 2018).

The physicochemical characteristics of these materials can be investigated through various analytical techniques. Fourier transform infrared spectroscopy (FTIR) is used to identify the functional groups of biological nanomaterials, X-ray diffraction (XRD) is employed to determine their crystal structures, and scanning electron microscopy (SEM) and transmission electron microscopy (TEM) are utilized to examine their morphology and size.

#### 3.4.1. The role of biological nanomaterials in wastewater treatment technology

Biological nanomaterials have increasingly become vital components of WWT due to their unique physicochemical properties, environmental compatibility, and high functionality. Biological nanomaterials offer economic advantages as they require less energy and fewer reagents than chemical synthesis methods, and they possess additional benefits such as minimizing secondary pollution. Biological nanomaterials are integrated into WWT and applied in the following areas:

- Nanofiltration and membrane systems: Nanoparticles enhance water quality by modifying membrane surfaces.
- Bioreactor systems: Nanoparticles enhance the activity of microorganisms and facilitate the removal of organic and inorganic pollutants.
- Photocatalytic systems: Nanoparticles accelerate pollutant degradation by utilizing light energy.

## 4. Applications of green nanomaterials in wastewater treatment technology

Different green templates—such as plant extracts, bacteria, and algae—are employed for the synthesis and characterization of nanoparticles and nanomaterials. This review addresses several treatment methods, including adsorption, catalytic oxidation of organic pollutants, and the use of photogenerated charges induced by irradiation. Green nanomaterials (GNMs) eliminate the use of hazardous chemicals and reduce troublesome waste generation, which are common in conventional 0D, 1D, and 2D nanomaterial preparation procedures in both top-down and bottom-up approaches. By utilizing naturally occurring bioreducing agents found in biological systems, GNMs can be synthesized without the use of hazardous compounds. Green nanoparticles have emerged as important components of WWT technology due to their unique features, environmental compatibility, and high efficacy in pollutant remediation. Their synthesis frequently involves biological components, yielding more stable and environmentally compatible products than traditional processes (Soni et al., 2021). Green synthesis approaches, such as those employing plant extracts and microbial processes, align with sustainability principles by reducing reliance on harmful chemicals and providing biodegradable alternatives (Kirubakaran et al., 2025). In WWT, green nanomaterials are primarily applied for heavy metal remediation, organic pollutant degradation, and pathogen inactivation (Kumar, 2021). For instance, ZnO NPs and their composites can effectively degrade dyes under UV light, demonstrating high efficiency in photodegradation processes (Thiam et al., 2025). Table 4 presents examples of applications reported in the past 5 years.

In WWT facilities, cost-effectiveness remains a crucial factor when using green nanomaterials. Biosynthesis techniques tend to reduce costs associated with complex procedures and the use of harmful chemicals. Furthermore, these materials are often derived from waste products or agricultural residues, making them economically viable and environmentally sustainable alternatives. Additional benefits include biocompatibility, reduced toxicity, and enhanced effectiveness in various environmental remediation applications (Singh et al., 2024).

Despite these advantages, the application of green nanomaterials faces several challenges. The size and morphology of nanomaterials may vary due to biological factors involved in their synthesis, potentially affecting performance consistency (Kirubakaran et al., 2025). Comprehensive toxicological studies are also required to evaluate the long-term environmental impacts of these materials. Moreover, the scalability of green synthesis methods for industrial applications remains a challenge, necessitating further research to develop standardized methods that ensure reproducibility and efficacy at larger scales (Nwuzor, 2024). Currently, the green synthesis of nanoscale metals lacks standardized production guidelines regarding mass balance and stoichiometric ratios, particularly from the perspective of industrial-scale production.

### 4.1. Plant-based nanomaterials

Plants are rich in biomolecules, including proteins, polysaccharides, and phytochemicals, which act as stabilizing, capping, and reducing agents in the environmentally friendly synthesis of nanoparticles. Seeds, roots, flowers, peels, leaves, fruits, and stem barks from numerous plant species have been studied as potential sources for NP synthesis. However, due to the presence of hydroxyl and ketone groups capable of binding to metals and exhibiting chelation, the type, concentration, and source of phytochemicals in a plant extract—as well as the extraction process—are known to influence the properties of the resulting nanostructure (Nobahar et al., 2021). Table 5 highlights publications from the past 5 years on green-synthesized nanoparticles derived from plant extracts.

### 4.2. Factors influencing the green synthesis of nanoparticles and their characterization techniques

**Figure 3:**
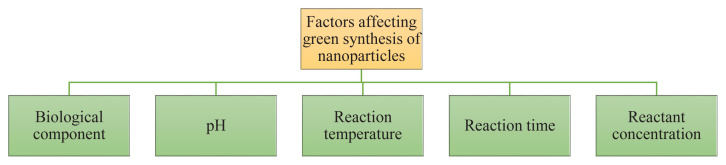
Factors influencing the green synthesis of nanoparticles.

Several parameters influence the efficiency and properties of this synthesis process, including the type of extract used, pH, temperature, light irradiation, and reaction time. Each of these parameters is crucial in determining the size, shape, and stability of the synthesized nanoparticles (see Figure 3). The phytochemical composition of the extracts significantly affects the reduction and stabilization processes. Plants contain varying levels of reducing agents, including phenolics, flavonoids, and terpenoids, which may influence nanoparticle formation and properties. The pH at which nanoparticles are synthesized determines their shape and size. For instance, Velgosová et al. (2016) reported that in acidic media, larger Ag NPs were formed, whereas in alkaline conditions, smaller and more uniform Ag NPs were produced. Aliannezhadi et al. (2025) reported that ZnO NPs synthesized at pH 11 exhibited a higher BET-specific surface area compared to other pH conditions, indicating enhanced photocatalytic activity.

The size and yield of NPs can be influenced by the duration of the reaction. Darroudi et al. conducted a study to investigate the effect of reaction time on particle size. Additionally, TEM analysis was performed on materials synthesized at different reaction times. The findings demonstrated that samples synthesized over extended reaction times exhibited a narrower particle size distribution (Darroudi et al., 2011). Therefore, careful optimization of reaction time is required to balance yield and desirable nanoparticle properties.

It has been shown that using light, particularly sunlight or LEDs, can significantly enhance the synthesis of nanoparticles. Light energizes the chemical mechanism, and research indicates that blue LED light is more effective than other wavelengths in enhancing silver ion bioreduction (Gurung et al., 2024) This demonstrates how photochemical processes can act as catalysts for green synthesis, potentially enabling the faster and more controlled production of nanoscale materials.

**Figure 4:**
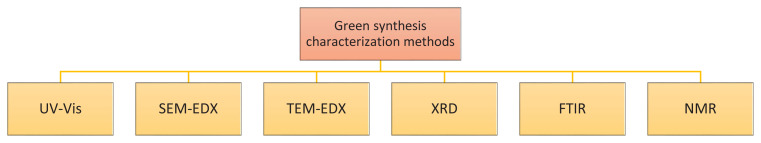
Characterization methods for nanoparticles synthesized via the green route.

Techniques such as TEM, SEM, and XRD are routinely employed for the characterization of green-synthesized nanoparticles (Figure 4). FTIR identifies the functional groups and bonding structures of nanoparticles; SEM and TEM determine their size and morphology at different magnifications; UV–Vis spectroscopy evaluates their stability; and XRD reveals their crystal structure. EDX analysis further confirms the elemental composition of the synthesized materials.

## 5. Challenges, limitations, and future perspectives

The technologies used in wastewater treatment are critically important in terms of environmental impact, energy consumption, efficiency, and cost. In this context, nanotechnology has the potential to serve as an alternative to conventional methods (Sati et al., 2022). Due to their small particle size, large surface area, high reactivity, rapid dissolution rate, and strong sorption capacity, nanomaterials offer effective solutions for pollutant removal. However, these same properties also pose risks of accumulation and toxicity to humans and aquatic organisms, depending on environmental conditions (Jain et al., 2021). The major limitation in wastewater treatment processes involving metal and metal oxide nanoparticles is the management of nanomaterials accumulated in sludge during treatment. Nanoparticles such as Mg, Ni, Ag, and Cd are known to accumulate in wastewater sludge, and the disposal of this accumulated material remains a major concern. Residual nanoparticle concentrations pose a serious problem, particularly in large-scale wastewater treatment plants (Olawade et al., 2024). Limitations in the use of carbon nanotubes (CNTs) in wastewater treatment include their disposable nature, small particle size, and the difficulty of separating them from sludge. Surface modification of carbon nanotubes can improve both their treatment efficiency and ease of disposal. For example, magnetically modified carbon nanotubes can be easily seperated from wastewater (Younas et al., 2021). Composite nanoparticles are synthesized from a wide range of materials and are increasingly being integrated into WWT studies. However, the major concern remains the lack of comprehensive safety analyses and nanotoxicity assessments. Many metal, metal oxide, carbon, and polymer-based nanomaterials can accumulate in human cells and exhibit toxic effects (Sonawane et al., 2021).

Biological and green nanomaterials are derived from natural resources through biological processes or green synthesis methods, offering nontoxic and environmentally friendly solutions. Consequently, they have shown significant progress in various fields. Green nanomaterials represent environmentally friendly, low-cost, and safe alternatives for wastewater treatment. However, transferring laboratory- and pilot-scale achievements to industrial applications poses challenges related to scalability, cost, stability, technology, and environmental impacts. The size and shape heterogeneity of biologically synthesized nanomaterials complicates commercial production and necessitates comprehensive toxicity assessments. Furthermore, the use of low-cost natural waste materials in nanomaterial production provides economic advantages while contributing to waste recycling (Paradva et al., 2023).

Biological and green nanomaterials have significant potential for application in various WWT facilities. Green nanomaterials are synthesized using environmentally friendly methods, minimizing potential adverse environmental impacts. Nevertheless, further research is required to assess the long-term environmental impacts of exposure to these materials. Future research should focus on topics such as production commercialization, recyclability, adsorption capacity, nanomaterial efficiency, and the selection of suitable and sustainable raw materials. Laboratory-scale studies are expected to be translated into industrial applications in the near future. In addition to commercialization efforts, toxicological analyses and life cycle assessments are required to evaluate environmental impacts from raw material acquisition to end use. Biological and green nanoparticles are expected to remain the focus of future studies as biocompatible, sustainable, nontoxic, and cost-effective solutions for WWT, considering the environmental challenges and costs associated with conventional methods and other nanomaterials. Therefore, researchers are expected to develop more effective and well-defined nanoparticles, optimize process parameters to enhance efficiency, and integrate novel nanomaterials into treatment systems to further improve the performance of green nanomaterials in WWT.

## 6. Conclusion

This review aimed to provide detailed information on recent studies related to wastewater treatment using biological and green nanomaterials. Biological and green-synthesized nanomaterials offer environmentally friendly, renewable, and economically sustainable solutions for wastewater treatment. These materials demonstrate effective adsorption and separation performance against various pollutants, primarily owing to their high surface area, rich functional group content, and biocompatible structures. Their high efficiency in removing dyes, heavy metals, and various organic pollutants has established these materials as a strong alternative to conventional methods.

However, several challenges must be addressed before these technologies can be widely implemented. These challenges include the high production cost of nanomaterials, the scalability of processes, integration into existing treatment systems, long-term stability, and reusability. Moreover, evaluating the environmental impacts of wastes generated after treatment and developing recovery strategies are of critical importance.

Future research should focus on diversifying biological sources for nanomaterial synthesis, standardizing synthesis protocols, and conducting comprehensive analyses of the environmental safety and reliability of these materials. Furthermore, translating laboratory-scale successes into pilot- and industrial-scale applications should be supported through multidisciplinary approaches and collaborations between the public and private sectors. In this context, innovative treatment technologies based on biological and green nanomaterials will play a pivotal role in achieving sustainable water management goals.

## Supplementary Information



## Figures and Tables

**Table 1:** Comparison of the properties of green and biological nanomaterials used in wastewater treatment technologies.

Key property	Green nanomaterials	Biological nanomaterials
Synthesis method	Derived from renewable resources through environmentally friendly synthesis methods	Produced through biological processes; however, the synthesis method may not always be environmentally friendly
Toxicity level	Nontoxic and biocompatible	Biocompatible and biodegradable
Surface area	High surface area, enabling efficient contaminant removal	High surface area, particularly in structures such as nanocellulose
Environmental impact	Minimal or no environmental impact due to their eco-friendly synthesis routes	Minimal environmental impact, as they are biodegradable and derived from natural sources
Distinctive properties	Catalytic, magnetic, and antibacterial properties	Self-assembly, biodegradability, and support microbial activity
Applications in wastewater treatment	Heavy metal removal, dye degradation, antibacterial treatment, and the degradation of organic pollutants	Bioremediation, nutrient removal, and nanofiltration membrane systems
Representative examples	Green-synthesized metal nanoparticles (e.g., silver), carbon nanotubes, and TiO_2_ nanoparticles	Bacterial cellulose, exopolysaccharides, and chitosan-based nanomaterials

**Table 2:** Biological nanomaterials commonly employed in wastewater treatment processes.

Biological nanomaterial	Source of origin	Area of application	Target pollutants
Nanocellulose	Plant fibers, bacterial cellulose	Adsorbent, membrane applications	Heavy metals, dyes
Nanochitin / nanochitosan	Shellfish exoskeletons	Adsorbent, biofilm carrier	Heavy metals, dyes
Nanoalginate	Brown seaweed	Nutrient (N, P) removal, microencapsulation	Ammonia, phosphate, heavy metals, dyes
Biologically synthesized Ag nanoparticles	Bacteria	Antimicrobial agent, microbial contamination control	*E. coli, Salmonella*, viruses
Biologically synthesized Fe_3_O_4_ nanoparticles	Microorganisms	Adsorbent, easily recoverable	Heavy metals
ZnO nanoparticles	Microorganisms / plant extracts	Photocatalytic degradation, microbial control	Organic pollutants, microorganisms
TiO_2_ biocomposites	Biopolymers + TiO_2_ nanoparticles	Photocatalyst	Dyes, pesticides
Bionanocomposites	Bioactive polymers + inorganic or metallic nanoparticles	All WWT processes	All pollutant groups

**Table 3:** Microorganism-based nanomaterials used in wastewater treatment.

Material	Nanoparticle type	Synthesized by	Particle size	Morphology	References
Algea	Ag NPs	*Sargassum muticum*	41.0 ± 5.7 nm	spherical	(González-Ballesteros et al., 2020)
	Au NPs	*Sargassum muticum*	10.4 ± 1.2 nm	spherical	(González-Ballesteros et al., 2020)
	Au NPs	*Chaetomorpha linum*	70–80 nm	spherical	(Acharya et al., 2021)
	CuO NPs	brown alga *Cystoseira trinodis*	6–7.8 nm	spherical	(Gu et al., 2018)
	ZnO NPs	*Spirogyra*	65 nm	spherical	(Hameed et al., 2023)
Fungi	ZnO NPs	endophytic fungus *Alternaria tenuissima*	15.45 nm	spherical	(Abdelhakim et al., 2020)
	Ag NPs	*Aspergillus terreus*	20 nm	spherical	(Li et al., 2012)
	Cu NPs	*Aspergillus niger.*	500 nm	round	(Noor et al., 2020)
	ZnO and Au NPs	*Endophytic fungus*	9.3 nm / 22.1 nm	spherical	(Hammad et al., 2025)
Bacteria	ZnO NPs	*Pseudomonas aeruginosa*	14.95 ± 3.5 nm	spherical	(Abdo et al., 2021)
	AuO NPs	*Pseudomonas aeruginosa*	15–30 nm.	n.d.	(Husseiny et al., 2007)
	AgO NPs	*Pseudomonas sp.*	10–40 nm	spherical	(Singh et al., 2018)

**Table 4:** Plant-based nanomaterials synthesized for wastewater treatment applications.

Nanomaterial	Plant	Size	Shape	References
rGO	*T. cordifolia*	30 nm	n.d.	(Saini et al., 2024)
CuO NPs	Lemon peel extract	34 nm	sphere-like morphology	(Kir et al., 2024)
Ag NPs	*Naringi crenulata*	32.75 nm.	Spherical to oval	(Chinnathambi et al., 2023)
NiO NPs	Cactus plant extract	20–35 nm	spherical	(Gebretinsae et al., 2021)
CdO NPs	*Citrus limetta*	51.5 nm	quasi-spherical	(Pagar et al., 2023)
CoO NPs	*P. guajava*	30.9 nm	sphere-shaped	(Govindasamy et al., 2022)
α-MoO_3_ NPs	*Tridax procumbens* leaf extract	n.d.	nanorods	(Aswin et al., 2025)
CeO_2_ NPs	*Morinda citrifolia* L.	29.2 nm	spherical	(Sukumaran et al., 2024)

**Table 5:** Application methods involving various green-synthesized nanoparticles.

Green Synthesis NPs	Biological source	Application method	Waste type	Experimental conditions	References
CuO NPs	*Seriphidium oliverianum* extract	Photocatalytic degradation	65% removal of MG and MO dyes	10 ppm dye concentration, 10 mg NPs, sunlight exposure for 60 min	(Aroob et al., 2023)
ZnO NPs	*P. roebelenii* leaf extract	Photocatalytic degradation	98% removal of MB dye in 105 min	250 W UV lamp, 10 ppm concentration, 0.2 g NPs	(Aldeen et al., 2022)
CuO NPs	*Eucalyptus globulus* leaf extract	Adsorption	92.5 mg/g adsorption capacity for MO at 25 °C	45 ppm concentration, 0.045 g adsorbent, pH 6.5	(Alhalili, 2022)
Ag/MgO NPs	*Acalypha hispida* extract.	Photocatalytic degradation	MB, 4-NP, MO, and 2,4-dinitrophenylhydrazine (2,4-DNPH)	0.03 mM MO, 0.031 mM MB, 10 mg catalyst	(Nasrollahzadeh et al., 2020)
TiO_2_ NPs	*Jatropha curcas* L. extract	Photocatalytic reduction	76.48% removal of Cr(VI)	300 min, 585 W/m^2^ irradiance, flow rate 0.25 L/s	(Goutam et al., 2018)
Fe and Fe/Pd NPs	green tea extract	Membrane-assisted degradation	Trichloroethylene	30 ppm TCE, 3.2 cm^2^ membrane area, 125 μm thickness	(Smuleac et al., 2011)
CuO NPs	*Catharanthus roseus* leaf extract	ultrafiltration membrane process	88.08% removal of CR(VI)	2 h, 5 ppm concentration	(Choudhury et al., 2018)
ZnO NPs	*Salvia officinalis* leaf extract	Photocatalytic degradation	92.47% MO degradation efficiency	500 W mercury lamp, 5 ppm dye concentration, 40 mg NPs, 1 h	(Abomuti et al., 2021)
