# Supplementary material for: Application of biological and green nanomaterials in wastewater treatment: techniques for the effective removal of dyes, heavy metals, and organic pollutants
Source: Turk J Biol. 2025 Aug 27;49(5):441–58. doi: 10.55730/1300-0152.2760 (PMC12614365; doi:10.55730/1300-0152.2760)
Supplement: Supplementary file 1 [file SI-TJOB.docx]

**Application of biological and green nanomaterials in wastewater treatment: techniques for effective removal of dyes, heavy metals and organics**

Emine Sena KAZAN-KAYA^1*^, Zeynep CİĞEROĞLU^2^, Tansel KEMERLİ KALBARAN^1^, Başak TEMUR ERGAN^1^, Zeynep Mine ŞENOL^3^, Meral YILDIRIM YALÇIN^4^

^1^Department of Chemical Engineering, Faculty of Engineering, Gebze Technical University, Kocaeli, Türkiye

^2^Department of Chemical Engineering, Faculty of Engineering, Uşak University, Uşak, Türkiye

^3^Department of Nutrition and Diet, Faculty of Health Sciences, Sivas Cumhuriyet University, Sivas, Türkiye

^4^Department of Food Engineering, Faculty of Engineering, Istanbul Aydin University, Istanbul, Türkiye

^*^Corresponding author:

Dr. Emine Sena Kazan-Kaya, [eskazan@gtu.edu.tr](mailto:eskazan@gtu.edu.tr)

ORCIDs:

First AUTHOR: https://orcid.org/ 0000-0002-0487-1984

Second AUTHOR: https://orcid.org/0000-0001-5625-6222

Third AUTHOR: https://orcid.org/0000-0003-2601-9968

Forth AUTHOR: https://orcid.org/0000-0002-0708-5102

Fifth AUTHOR: https://orcid.org/0000-0002-5250-1267

Sixth AUTHOR: <https://orcid.org/0000-0002-5885-8849>

**Table S1.** Advantages and disadvantages of common wastewater treatment techniques and their industrial applications (Crini & Lichtfouse, 2019; Kato & Kansha, 2024; Younas et al., 2021).

| Treatment Techniques | Industrial Application | Advantages | Disadvantages |
| --- | --- | --- | --- |
| Physical Treatment | | | |
| Sedimentation | Cement industry  Oil/Petroleum industry  Pulp and paper industry | - Does not require any energy input. - Demonstrates outstanding reproducibility. - Low cost and simple to design and operate. | - Selective process. - Long retention time is necessary for settling fine particles - May lead to large tank volume - Difficulty in removing very fine particles and dissolved content |
| Filtration (membrane filtration, ultrafiltration, nanofiltration, reverse osmosis etc.) | Ceramic industry  Food industry  Iron and steel industry  Textile industry  Pulp and paper industry  Oil/Petroleum industry | - No chemicals required - Simple, rapid and effective at high concentrations - Generation of low solid waste - Small space requirement - Production of a high-quality-treated effluent | - Time consuming process - Filters may become obstructed - Limited flow rates - Too high investment costs for small and medium industry - High maintenance and operation cost - Less effective when solute concentrations in the feed are low - Susceptibility to fouling |
| Ion Exchange | Textile industry  Chemical industry | - Rapid process - Small space requirement - Possibility of regeneration of resin - Technologically simple - Easy control, easy usage and maintenance - Production of a high-quality treated effluent - Suitable for various flow conditions, including continuous and batch processes. | - Economic challenges (maintenance cost, initial cost of selective resin, time-consuming) - Restricted selectivity of conventional resins - Pretreatment requirement of the most effluents - Ionic competition - Reactors quickly reach saturation and are prone to clogging. - Requirement of chemical regeneration |
| Adsorption | Oil/Petroleum industry  Ceramic industry  Chemical industry  Food industry  Iron and steel industry | - Low operational cost - Technologically simple design - Non-toxic process - An efficient process with rapid kinetics - High quality of the treated effluent - Usage for high variety of target contaminants | - Low selectivity of adsorbent - Requirement for several types of adsorbents - Reactors quickly reach saturation and are prone to clogging. - Not financially feasible for specific industries (pulp and paper, textile etc.) |
| Degasification | Oil/Petroleum industry  Pulp and paper industry | - Cost efficient technique - Reduces the need for additional chemical usage in subsequent treatment processes. | - Has a restricted ability to eliminate pollutants effectively. |
| Chemical Treatment | | | |
| Coagulation/Flocculation | Cement, concrete, and ceramics industry  Chemical industry  Food industry  Iron and steel industry  Pulp and paper industry  Textile industry | - Possible usage of fine particle removal - Suitable for fine particle removal - Eliminates metals, discoloration, and turbidity. - Operational simplicity - Substantial decrease in chemical oxygen demand (COD) and biochemical oxygen demand (BOD). - Fast and effective in removing insoluble contaminants such as pigments. | - High operational cost resulting from chemical usage - Higher production of sludge and sludge disposal cost - A multi-step process - Harmful if not handled correctly. - Limited efficiency in arsenic removal. |
| Chemical Precipitation | Pulp and paper industry | - Technologically simple and manageable process operation - Low operational cost - Effective over a wide temperature range - Suitable for handling high concentrations of pollutants. - Highly effective in removing metals and fluoride. - Considerable decrease/reduction in the chemical oxygen demand | - Production of large quantity of sludge and additional operational cost for sludge management. - Sludge disposal is difficult. - The metal ions are not effectively removed at low concentration. - Use of chemicals (lime, oxidants, H_2_S etc.) |
| Solvent Extraction | Oil (Petroleum) industry  Pharmaceutical industry  Wool industry | - A well-known, proven method of wastewater recycling separation - Easy process control and monitoring, low operational cost - Effective for phenol separation - An effective substitute for classical lime precipitation in the recovery of phosphoric acid | - High investment (equipment) - Not cost-effective at low contaminant concentrations (<0.5 g/L) - Use of large amounts of organic extractants and potentially hazardous solvents. - Limitations of hydrodynamics (entrainment and flooding) - Phase entrainment resulting in low effluent quality - Phase emulsification accompanied by poor separation - Possible effects of solvent use on health and the environment. |
| Electrochemical Methods (Electrochemical oxidation (EO), Electroreduction (ER), Electrodialysis (ED), Electrocoagulation (EC), Electroflocculation (EFC) and Electroflotation (EF) etc.) | - Food, textile, petroleum, pulp and paper, and pharmaceutical industries (EC) - Petroleum, pulp and paper, and pharmaceutical industries (EO) - Miming industry (EFC) - Iron and steel industry (EO) | - Simple operation, low sludge generation, no chemical addition, and efficient removal of colloids and reduction, coagulation and separation of copper (EC) - Reduced secondary pollution, minimal environmental impact, no chemical addition, and the capacity to degrade recalcitrant pollutants (EO) - Metal recovery and removal, minimal environmental impact, no chemical addition, and the transformation of pollutants into materials with added value (ER). - Low operating pressures, minimal scaling susceptibility, high salt and metal removal, and no chemical addition (ED) | - Power consumption, electrode fouling, the potential for metal hydroxide dissolution, and routine sacrificial anode replacement (EC) - Excessive energy use, the high cost of some electrodes (like those made of diamond and noble metals), difficulties in mass producing some electrodes, the potential for electrode fouling and corrosion, and electrode replacement (EO). - High energy consumption, the need to manage competition with the hydrogen evolution reaction, the potential for some corrosion, and the high cost of noble-metal electrodes (ER). - Membrane fouling, excessive energy and capital costs, and the incapacity to eliminate nonionic pollutants (ED) - The effectiveness of separation is highly dependent on bubble sizes (EFC). |
| Chemical Oxidation | Pulp and paper industry  Textile industry  Chemical industry  Food industry | - A quick, easy, and effective method - Odor and color removal, disinfection (bacteria and viruses) - Efficiency and adaptability in eliminating a variety of organic pollutants - A shorter treatment contact period than biological approaches | - Effects of the oxidizing agent used and the intermediates created on the environment - Production and storage-related operating costs - Transport, oxidant use, and the necessity of pretreatment |
| Advanced Oxidation Processes (O_3_/H_2_O_2_/UV Oxidation, Fenton oxidation (FO), Photocatalysis (PC), Sonolysis (S) | - Textile pharmaceutical, petroleum, and aquaculture industries (O_3_/H_2_O_2_/UV Oxidation) - Oil, textile, pharmaceutical and pulp and paper industry (FO) - Chemical industry, iron and steel industry, pharmaceutical industry, pulp and paper industry, textile industry (PC) | - Complete mineralization, disinfection, no production of halogenated byproducts, nonselective oxidation of pollutants by radical species, ease of use, and the ability to reuse treated wastewater (O_3_/H_2_O_2_/UV Oxidation). - Ease of implementation, efficient breakdown of pollutants, availability of Fe²⁺ and H₂O₂, and environmentally friendly characteristics (FO). - Broad-spectrum degradation and mineralization of various organic compounds, minimal chemical usage due to the reusable photocatalyst, and the potential to utilize solar irradiation (PC). - Safety, environmental sustainability, no need chemical, effectiveness as a pre-treatment to improve biodegradability, and capability to break down persistent organic compounds (S). | - Investment and operational costs associated with ozone generation, UV irradiation, and hydrogen peroxide application, along with the safety risks involved in handling ozone and hydrogen peroxide (O_3_/H_2_O_2_/UV Oxidation). - Requirement for managing sludge disposal, pH level control, and utilizing large quantities of chemicals (FO). - Requirement for effective light irradiation of the photocatalyst, operational expenses, energy input of artificial light sources, challenges related to photocatalyst fouling and instability, safety risks associated with certain photocatalysts, the necessity for photocatalyst recovery in slurry reactors, and the overall cost of equipment (PC). - Cost of equipment, significant energy requirements, and the transformation of cavitational energy into chemical and physical effects (S). |
| Biological Treatment | | | |
| Aerobic processes (activated sludge (AS), membrane bioreactor (MBR), sequential batch reactor (SBR), trickling filter (TF), aerated lagoons (AL)) | Chemical Industry  Iron and steel Industry  Pharmaceutical Industry  Pulp and paper industry  Food industry  Textile industry | - Low footprint, high effluent quality, and inexpensive installation costs and High removal of suspended solids and biochemical oxygen demand (AS). - Controllable, flexibility, small footprint, cost efficiency due to the ability to perform multiple unit operations within a single tank (SBR). - Simplicity, reliability, and low power consumption (TF). - High-quality effluent generated by membranes, compact size, and ease of automation (MBR). - Less land usage than stabilization ponds, less sludge generation than other secondary treatment methods, and lower operation and management costs than activated sludge (AL). | - Poor decolorization, high operational costs, sludge bulking and foaming, and sensitivity to effluent characteristics (AS). - Complicated maintenance, operation, and potential blockages and sludge discharge in aeration equipment (SBR). - Odor emission and the requirement for extra care and operator supervision (TF). - Fouling, the requirement to maintain membranes, foaming, excessive electricity consumption, and high operating and capital expenses (MBR). - lower nutrient removal than stabilization ponds, higher land usage than activated sludge, and higher energy input for aeration (AL). |
| Anaerobic processes (Upflow anaerobic sludge blanket, expanded granular sludge bed) | Chemical Industry  Iron and steel Industry  Pharmaceutical Industry  Textile industry | - The advantages of anaerobic reactors include efficient chemical oxygen demand removal at high organic loadings, biogas production, low energy demand and operational costs, a small footprint, lower sludge production than AS, and generation of biologically stabilized sludge. | - Temperature sensitivity, sludge granulation is difficult to control, depends on wastewater quality, requires pretreatment to remove total suspended solids (TSS), and is sensitive to shock loads. |

**References**

Crini G, Lichtfouse E (2019). Advantages and disadvantages of techniques used for wastewater treatment. Environmental Chemistry Letters 17(1): 145–155. https://doi.org/10.1007/s10311-018-0785-9

Kato S, Kansha Y (2024). Comprehensive review of industrial wastewater treatment techniques. Environmental Science and Pollution Research 31(39): 51064–51097. https://doi.org/10.1007/s11356-024-34584-0

Younas F, Mustafa A, Farooqi ZU, Wang X, Younas S, Mohy-Ud-Din W, Ashir Hameed M, Mohsin Abrar M, Maitlo AA, Noreen S, Hussain MM (2021). Current and Emerging Adsorbent Technologies for Wastewater Treatment: Trends, Limitations, and Environmental Implications. https://doi.org/10.3390/w13020215
